# An objective structured clinical exam to measure intrinsic CanMEDS roles

**DOI:** 10.3402/meo.v21.31085

**Published:** 2016-09-15

**Authors:** Aliya Kassam, Michèle Cowan, Tyrone Donnon

**Affiliations:** 1 Office of Postgraduate Medical Education, Department of Community Health Sciences, Cumming School of Medicine, University of Calgary, Calgary, AB, Canada; 2 Office of Postgraduate Medical Education, Cumming School of Medicine, University of Calgary, Calgary, AB, Canada; 3 Department of Community Health Sciences, Cumming School of Medicine, University of Calgary, Calgary, AB, Canada; 4 Testing & Measurement, Canada's Testing Company, Assessment Strategies Inc., Ottawa, ON, Canada

**Keywords:** postgraduate medical education, competency-based medical education, objective structured clinical exam, intrinsic roles

## Abstract

**Background:**

The CanMEDS roles provide a comprehensive framework to organize competency-based curricula; however, there is a challenge in finding feasible, valid, and reliable assessment methods to measure intrinsic roles such as *Communicator* and *Collaborator*. The objective structured clinical exam (OSCE) is more commonly used in postgraduate medical education for the assessment of clinical skills beyond medical expertise.

**Method:**

We developed the CanMEDS In-Training Exam (CITE), a six-station OSCE designed to assess two different CanMEDS roles (one primary and one secondary) and general communication skills at each station. Correlation coefficients were computed for CanMEDS roles within and between stations, and for general communication, global rating, and total scores. One-way analysis of variance (ANOVA) was used to investigate differences between year of residency, sex, and the type of residency program.

**Results:**

In total, 63 residents participated in the CITE; 40 residents (63%) were from internal medicine programs, whereas the remaining 23 (37%) were pursuing other specialties. There was satisfactory internal consistency for all stations, and the total scores of the stations were strongly correlated with the global scores *r*=0.86, *p*<0.05. Noninternal medicine residents scored higher in terms of the *Professional* competency overall, whereas internal medicine residents scored significantly higher in the *Collaborator* competency overall.

**Discussion:**

The OSCE checklists developed for the assessment of intrinsic CanMEDS roles were functional, but the specific items within stations required more uniformity to be used between stations. More generic types of checklists may also improve correlations across stations.

**Conclusion:**

An OSCE measuring intrinsic competence is feasible; however, further development of our cases and checklists is needed. We provide a model of how to develop an OSCE to measure intrinsic CanMEDS roles that educators may adopt as residency programs move into competency-based medical education.

The Royal College of Physicians and Surgeons of Canada (RCPSC) introduced the CanMEDS roles framework as the basis for the development of medical curricula and measurement methods to assess resident physicians (RPs) throughout their training programs (1, 2). This framework situates the *Medical Expert* role centrally and integrates six other roles referred to as the intrinsic roles (*Communicator*, *Collaborator*, *Leader* (formally *Manager*), *Health Advocate*, *Scholar*, and *Professional*) to provide a comprehensive overview of the competencies expected of all physicians (1, 2).

Since the latest revision in 2015 (2–4), the RCPSC has championed the need for the development and integration of a competency-based medical education (CBME) approach for postgraduate medical education that includes the continuum of learning from residency to practice (5). Although the CanMEDS roles provide a comprehensive framework to organize competency-based curricula, the challenge has been to find feasible, valid, and reliable assessment methods to measure these diverse clinical competencies (6–8).

The objective structured clinical exam (OSCE) has been widely used in the postgraduate medical education (9–14). With the increased attention and demands for CBME, the standardized patient (SP)-based OSCEs are becoming more commonly used for assessment of various medical competencies.

A number of studies have looked at the use of the OSCE to assess specific skills within particular residency training programs. Jefferies and colleagues (13) found the use of the OSCE to be a reliable and valid method of simultaneously assessing the CanMEDS roles in physicians from neonatal and perinatal training programs with Cronbach's alpha values of 0.80–0.88 for internal consistency reliability. The knowledge and skills of postgraduate year 2–5 (PGY-2, 3, 4, 5) orthopedic residents have been used with success in an OSCE format as it allowed direct observation of the residents’ clinical skills development in a safe and controlled environment. It also allowed supervisors to monitor residents’ progress longitudinally throughout their training (12). Another orthopedics program, which examined intrinsic roles in 25 residents using an OSCE and an in-training evaluation report (ITER) data, showed a significant effect of PGY level across total test scores, individual station scores, and individual CanMEDS roles scores (15). Hybrid simulation OSCEs have also been used to assess CanMEDS roles in urologic residents where the overall scores for Communicator, Manager, and Health Advocate were similar across all of the residents. Procedural skills scores related to being a Medical Eexpert, however, were found to be higher for the senior residents (16). Another study found that the SP-based OSCE can be an effective method to assess communication and interpersonal skills of surgical residents in a clinical setting (17).

Although the OSCE has been shown as an effective method for evaluating competencies, the scores obtained from an OSCE can be vulnerable to potential measurement errors that cases, items, or SPs can introduce (10, 13, 18–20). Furthermore, none of the existing studies have used a framework of validity to ascertain the sources of validity evidence for the OSCE. Likewise, although CanMEDS roles framework has been shown to be useful in developing educational goals (21), a rigorous, reliable, and valid assessment of each competency often presents a challenge to training programs. For example, at our institution although there have been faculty development resources in teaching intrinsic roles such as Professional and Communicator, there is little guidance around assessing intrinsic role competence in residents other than that provided by the RCPSC. Given these scenarios, competencies are difficult to assess with knowledge-based written examinations; this necessitated the development of an assessment method, known as the CanMEDS In-Training Exam (CITE), for the intrinsic expert CanMEDS roles.

By examining the CanMEDS competencies of residents using an OSCE approach to testing, the focus was on assessing residents’ gaps in their training that related to the Communicator, Collaborator, Manager, Health Advocate, and Professional roles. The purpose of this study was to establish validity evidence for and determine the feasibility of an OSCE developed to measure intrinsic CanMEDs roles.

## Method

This study received ethical approval from the University of Calgary Research Ethics Board (REB ID# 24142). The Office of Postgraduate Medical Education organized the recruitment and selection of both junior and senior residents for the CITE. The CITE was designed to be used as a set of OSCE scenarios to measure a primary and secondary CanMEDS role and a generic assessment of the residents’ communication skills at each station. The CITE was also intended to be a formative assessment tool for residents across all programs and PGY levels; hence, this was a pilot test of its development, implementation to determine feasibility.

A maximum of 64 residents were invited to participate voluntarily in the CITE, which took place in March 2012 and was administered at the Medical Skills Centre facilities. There were two tracks with eight stations in each track for a morning session, consisting of 32 registrants in the morning session and 32 registrants in the afternoon session. Those residents who were not in a core internal medicine program or subspecialty in internal medicine (e.g., dermatology) were considered internal medicine residents, whereas those residents in other programs (e.g., surgical specialties, family medicine, pediatrics, and pathology) were considered noninternal medicine residents.

**Fig. 1:**
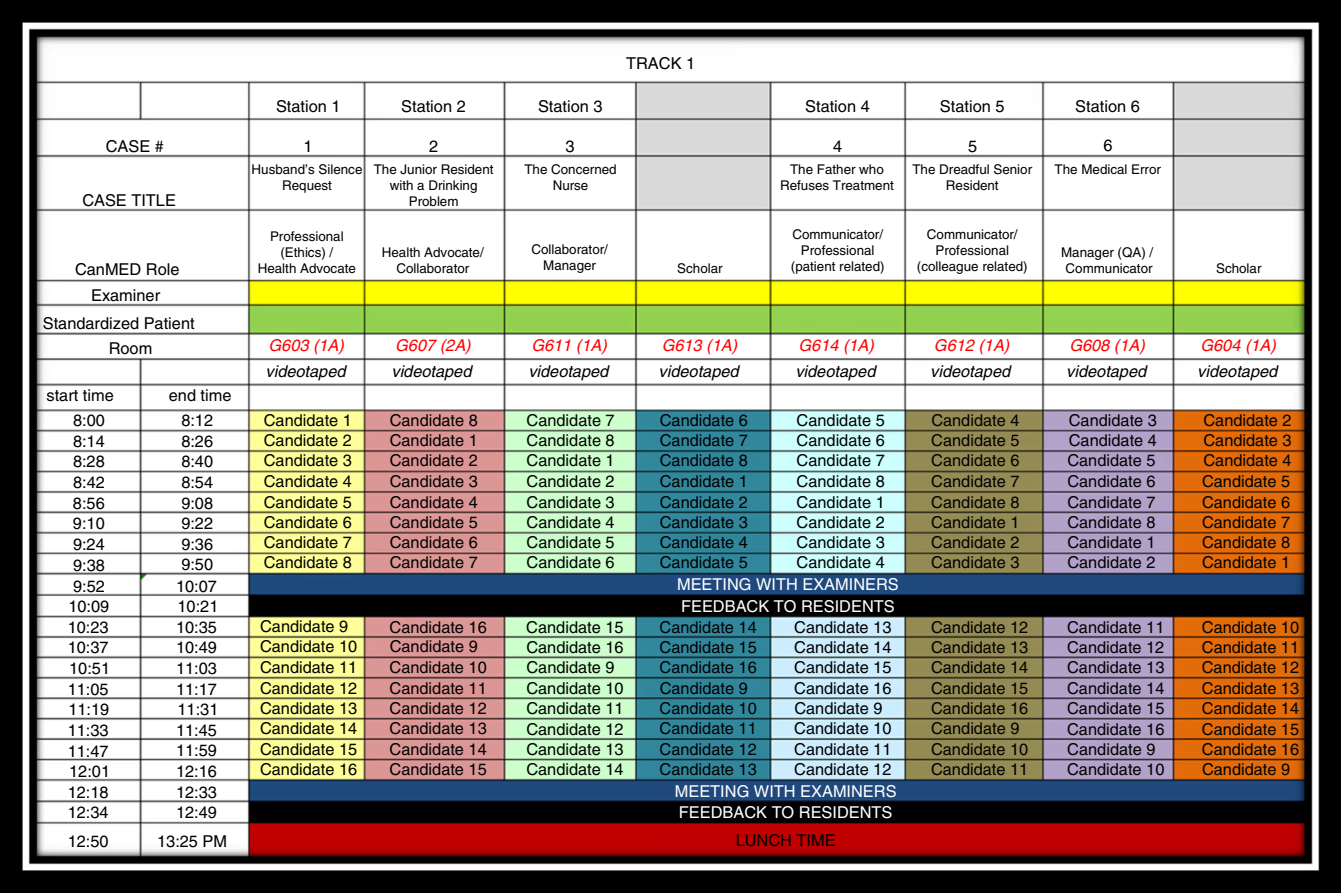
Example of Track 1 Schedule and CanMEDS roles assessed for the CITE OSCE stations.

Each OSCE scenario was allocated 10 min before residents were asked to move to the next station, and an additional 2 min was allowed for the change between stations. A 15-min feedback session was provided to all residents after they completed all eight stations. The entire exam, including the feedback session, took approximately 2.5 h. Additional personalized feedback pertaining to resident mean scores and overall mean scores was emailed to residents within a month of completing the OSCE. This paper describes the results from the six stations that included the intrinsic roles of Communicator, Collaborator, Manager, Health Advocate, and Professional. Two of the eight stations measured the Scholar role exclusively and have been described elsewhere (22). Figure 1 illustrates the OSCE administration schedule for track 1 (morning session) and outlines the CanMEDS roles being assessed at each of the stations.

### CITE case and checklist development

The development of each OSCE case scenario was based on the CanMEDS framework and was written to take into consideration two CanMEDS roles and their related competencies. Each case scenario is designed to contain a primary and secondary role. By ‘primary’, we meant that this particular role was the principal focus of the case and was measured using more detailed items in the checklist. By ‘secondary’, we meant that this role was supportive to the primary role but not the focal point of the case and was measured using fewer items on the checklist.

The Communicator role, with respect to the ability to develop rapport, accurately elicit and convey relevant information, was assessed generically for all cases, whereas more specific aspects of the Communicator role were measured in three OSCE stations as either a primary (stations 4 and 5) or secondary (station 6) role. In the development of the CITE, each intrinsic role was written into a case as a primary role at least once and in another case at least once as a secondary role. The rationale behind this was that it would have been too difficult to measure all of the CanMEDS roles at once. The specific primary and secondary designation of roles in each case was preset to facilitate the development of case writing and the corresponding checklist development that was used for the assessment of the residents.

The following is the list of the assigned CanMEDS competencies for each of the six cases and a brief description of the essence of the scenario:

- Professional – ethics (primary)/Health Advocate (secondary): *Husband's silence request* pertained to keeping information about wife's illness from her.
- Health Advocate (primary)/Collaborator (secondary): *Junior resident with a drinking problem* pertained to advocating for a colleague having self-care issues.
- Collaborator (primary)/Manager (secondary): *Concerned nurse* pertained to quality assurance issues on unit.
- Communicator (primary)/Professional – patient related (secondary): *Father who refuses treatment* pertained to a parent who refused treatment for child that was against the belief of the resident.
- Communicator (primary)/Professional – colleague related (secondary): *Dreadful senior resident* pertained to organization and time management issues of a colleague.
- Manager – quality improvement (primary)/Communicator (secondary): *Medical error* pertained to disclosure of an error and future plans to avoid such adverse events.

Expertise and feedback from clinicians were incorporated into the developmental process to ensure content validity of the checklist. Clinical expertise was necessary to confirm that the evaluation content was both relevant and practical. Where necessary, an SP or standardized health care provider was used during the exam. Instructions for SPs to follow were developed and the research team met with SPs/health care providers in advance of the exam to go through each scenario and answer any questions. SPs/health care providers were also provided with training on how to respond and react to the residents on their respective scenarios 3 weeks prior to the exam.

Checklists were developed with feedback from clinicians. Checklist items were intended to be case specific, incorporating the key competencies of each CanMEDS role (except *Scholar* and *Medical Expert*). The completed development of each case scenario and its competency checklist for the exam consisted of a two-step process, which involved:

1. Reviewing the case scenario examples provided and reviewing feedback to improve the case or suggestions for an alternative case and
2. Writing items for the corresponding checklist that would reflect competency expectations specific to the case which would be used in the evaluation of the residents.

Once checklists were developed, they were further revised by two of the authors (AK and TD), and a final review was completed by a clinician. Examiners were presented with their case 1 week prior to the exam to familiarize themselves with the case and checklist items. Examiners were recruited through program directors as candidates with previous examiner and preceptor experience were identified. Those examiners who were able to participate for the entire day observed a different case in the afternoon session to mitigate examiner fatigue.

### Validity assessment

When assessing the validity and reliability of the CITE, we used the framework adopted by the American Educational Research Association (AERA), American Psychological Association (APA), and the National Council on Measurement in Education (NCME) as a field standard (23). In this framework, all forms of validity are considered to be construct validity, and evidence of construct validity is collected from five different sources (24, 25). Construct validity is the appropriateness of inferences made on the basis of observations or measurements (in this case, the CITE OSCE checklist scores), specifically whether the OSCE measures the intended construct which would be intrinsic CanMEDS roles (23).

Reliability is also considered part of validity with respect to the internal structure of the checklists. The five sources of evidence sought to support or refute a validity argument under this framework include *content*, *response process*, *internal structure*, *relationships with other variables*, and *consequences*. The following is a brief description of each of the sources of validity evidence: *content evidence* – a series of measures taken to examine if the assessment content is representative of the intended measurement construct. This may consist of formulation based on prior instruments, seeking expert review, or utilizing an assessment framework.

*Response process evidence* – evaluation of how well the responses or raters’ actions relate with the intended measurement construct. This includes assessment security, quality control, and the analysis of the respondents’ thoughts and/or actions during assessment completion (24, 25).

*Internal structure evidence* – evaluation of how well assessment items align with the overall construct. This should include a measure of reliability across items or raters, but may also include item analysis or factor analysis (24, 25).

*Relations with other variables evidence* – statistical associations between the assessment rating and other measures or features that could influence or have a relationship (24, 25).

*Consequences evidence* – the result (beneficial or harmful) of the assessment, and the subsequent decisions or actions, which includes what distinguishes or influences such outcomes (24, 25).

This framework is useful because it provides a more comprehensive examination of validity in assessment, and also incorporates examination of reliability under *internal structure evidence*.

### Data collection

**Fig. 2:**
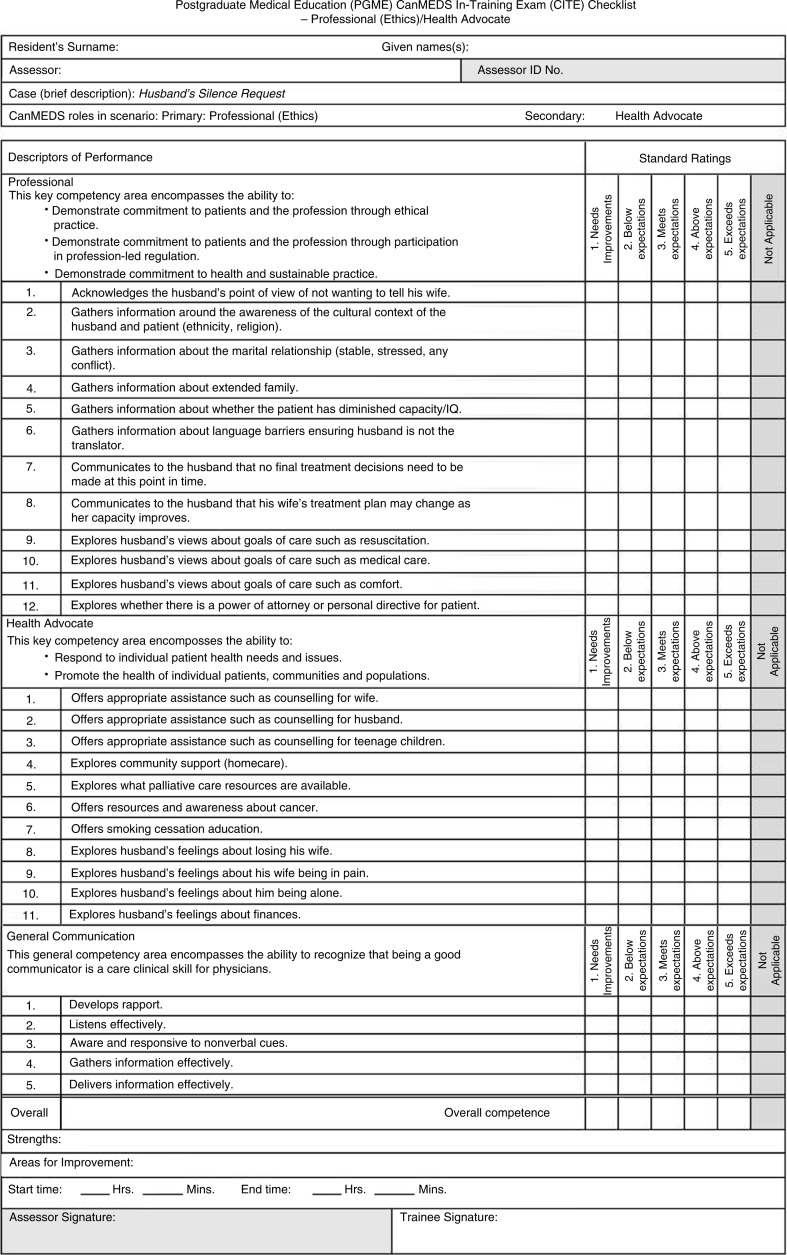
Example of checklist used in the CITE.

All residents signed a consent form prior to the CITE to have their OSCE video-recorded. There was one examiner present for each OSCE station. Hardcopy checklists were used during the exam, and each of the six station checklists had similar formats. Figure 2 shows an example of a checklist for the first case used in our OSCE.

Checklists for the six stations had 17–23 items, with the majority of the items measuring competence in the primary CanMEDS role; however, all had the same additional five items that measured general communication skills. These included items pertaining to developing rapport, listening effectively, responding to nonverbal cues, as well as gathering and delivering information effectively. As such, the total number of items for the checklists ranged from 22 to 28. Each checklist item was scored as 0 (not at all), 1 (minimally), or 2 (completely). For example, the score on a 22-item checklist would range from a minimum score of zero (0×22 items) to a maximum score of 44 (2×22 items). The overall competence (global rating scale score) of the resident at each OSCE station was measured from 1 (fails to meet expectations) to 5 (exceeds expectations).

### Data analysis

Data were inputted into a database and analyzed using STATA version 13.0. Within stations, correlation coefficients were calculated for the scores pertaining to the 1) primary CanMEDS role, 2) secondary role, and 3) the total score on the five communication items for each station. Total mean station scores were also computed and correlated across stations. Correlation coefficients were also computed between stations on the global rating scale scores, general communication scores, and for the CanMEDs roles assessed across all stations. Given the exploratory nature of this study, an alpha level of 0.05 was used.

The total mean scores for each checklist (primary role+secondary role+general communication) and mean global score were calculated and stratified by 1) noninternal medicine residents versus internal medicine residents, 2) senior residents versus junior residents, and 3) sex of the resident. Junior residents were classified as those persons at the PGY-1, 2, 3 levels in their respective residency programs, while senior residents were those at the PGY-4, 5 levels. Stata computed the Bartlett's test for equal variances, which allowed us to test the assumption of analysis of variance (ANOVA) that the variances were the same between groups. A small value for Bartlett's statistic confirmed that this assumption was not violated in our data and that the use of an ANOVA was acceptable. In cases where this assumption was violated, a *t*-test for groups with unequal variances was used.

## Results

Of the 64 spaces available for the CITE, 63 residents (98% response rate) participated and completed the testing requirements (one resident did not attend the OSCE). There were 31 males (49%) and 32 females (51%). Forty residents (63%) were internal medicine residents who were specializing either in internal medicine or in an internal medicine subspecialty, whereas the remaining 23 (37%) were pursuing other specialties. There were 21 (33%) junior residents and 42 (67%) senior residents. Table 1 shows the reliability coefficients (Cronbach's alpha) values for the full checklist (i.e., all checklist items including the primary and secondary CanMEDS roles, and the five general communication items), and the mean total and global rating scale scores for each station.

**Table 1:** Cronbach's alpha and total mean scores for each station (all checklist items including primary role, secondary role, and five communication items)

Case	Roles	Total checklist items (#)	Cronbach's alpha	Mean score (SD) range	95% C.I.	Mean global scale score, SD) range	95% C.I.
1	PRO/HA	28	0.75	22.4 (6.0) 11–39	20.8–23.9	2.9 (0.62) 1–5	2.7–3.0
2	HA/COL	23	0.83	32.6 (7.5) 14–46	30.8–34.5	3.2 (0.98) 1–5	2.9–3.4
3	COL/MAN	23	0.78	34.5 (5.6) 19–44	33.1–35.9	3.4 (0.87) 2–5	3.2–3.6
4	COM/PRO) (patient)	22	0.85	35.3 (6.4) 20–44	33.6–36.9	3.2 (1.01) 1–5	2.9–3.4
5	COM/PRO) (colleague)	22	0.76	30.4 (5.9) 16–44	28.9–31.9	2.9 (0.82) 1–5	2.7–3.1
6	MAN/COM	22	0.79	29.1 (6.0) 17.41	27.6–30.7	3.0 (0.76) 1–4	2.8–3.2

PRO=Professional; HA=Health Advocate; COL=Collaborator; COM=Communicator; MAN=Manager.

### Correlation coefficients

There were no clear patterns shown in the correlations between CanMEDS roles scores (primary and secondary). The total scores of all stations, however, were strongly correlated with total global scores (*r*=0.86, *p*<0.05).

In terms of total scores for each station, station 4, *Communicator* (primary), *Professional* – *patient related* (secondary) was significantly correlated with stations 1 (*r*=0.31, *p*<0.05), 2 (*r*=0.31, *p*<0.05), and 3 (*r*=0.27, *p*<0.05) but not stations 5 and 6 which did not include a direct patient component to the case.

General communication scores were strongly correlated with global scores, *r*=0.80, *p*<0.05. In terms of general communication, scores within each station were significantly correlated with the primary and secondary CanMEDS roles total scores in their respective station with correlation coefficients ranging from *r*=0.52 to 0.65, *p*<0.05.

General communication across stations did not show clear patterns. For example, station 1, *Manager* – *quality improvement* (primary)/Communicator (secondary), was negatively correlated with station 6 (*r*=0.40, *p*<0.003). Regarding total mean scores, station 6 was positively correlated with stations 3 (*r*=0.40, *p*<0.003) and 4 (*r*=0.42, *p*<0.003). When examining CanMEDS roles across all stations, the only significant correlation was between the total score for *Communicator* and the total score for *Health Advocate* (*r*=0.42, *p*<0.003).

When looking at station-specific CanMEDS roles scores, there were significant correlations between the primary and secondary roles (despite being different CanMEDS roles) within each station for all of the stations with correlation coefficients ranging from *r*=0.35 to 0.60, *p*<0.05.

For CanMEDS roles across stations, there were small correlations between CanMEDS roles (despite being primary or secondary) but no clear patterns emerged. For example, the *Health Advocate* CanMEDS role (secondary role) questions in station 1 were significantly correlated with the *Communicator* CanMEDS roles (primary role) questions in station 5 (*r*=0.38, *p*<0.003). Similarly, a negative correlation was found between the *Collaborator* CanMEDS role (primary role) in station 3 and the *Professional* (colleague related) CanMEDS role (secondary role) in station 5 (*r*=−0.53, *p*<0.003). The only significant moderate correlation found on the same CanMEDS role was between station 4 (primary role) and station 6 (secondary role) for *Communicator* (*r*=0.52, *p*<0.003).

### Analysis of variance

A one-way ANOVA did not show any significant differences between CanMEDS roles scores (primary and secondary), general communication scores, and total station scores for each station when stratified by the sex of the resident or year of residency. Regarding the CanMEDS role of *Professional* across all stations, senior residents scored significantly higher than junior residents 30.6 versus 28.0, *p*<0.05. There were significant differences on total mean scores found between internal medicine residents versus non-internal medicine residents at three stations (Table 2).

**Table 2:** Total mean (SD) scores for each of the CITE stations by internal medicine and other specialty residents

Case no.	CanMEDS roles measured (primary/secondary)	Internal medicine Total mean (SD) score	Other specialty Total mean (SD) score	ANOVA *p*
1	Professional – ethics/Health Advocate	20.9 (5.7)	24.9 (5.8)	0.01*
2	Health Advocate/Collaborator	33.2 (7.5)	31.8 (7.6)	0.49
3	Collaborator/Manager	36.0 (4.5)	31.9 (6.3)	0.004*
4	Communicator/Professional – patient related	35.8 (6.5)	34.4 (6.4)	0.41
5	Communicator/Professional – colleague related	29.1 (5.8)	32.8 (5.4)	0.01*
6	Manager – quality improvement/Communicator	30.1 (5.9)	27.4 (6.1)	0.09

* Significant at *p*<0.05.

There were also significant differences between internal medicine residents and non-internal medicine residents for the *Professional* and *Collaborator* CanMEDS roles when the same CanMEDS roles scores were tabulated across different stations. Noninternal medicine residents scored higher in terms of the *Professional* competency, whereas internal medicine residents scored higher in terms of the *Collaborator* professional competency (Table 3).

**Table 3:** Total mean (SD) scores for each of the CanMEDS roles for internal medicine and other specialty residents

CanMEDS role	Internal medicine Total mean (SD) score	Other specialty Total mean (SD) score	ANOVA *p*
Professional	28.9 (4.0)	31.2 (2.4)	0.006*
Health Advocate	17.0 (4.6)	16.9 (6.8)	0.93
Collaborator	30.8 (4.1)	27.9 (4.2)	0.01*
Communicator	35.0 (7.0)	35.1 (6.5)	0.95
Manager	22.8 (3.6)	22.2 (4.7)	0.55

* Significant at *p*<0.05.

## Discussion

The CITE study was designed to fit within the format of an OSCE method of assessment to measure 63 residents’ competencies in intrinsic CanMEDS roles, namely *Professional*, *Health Advocate*, *Communicator*, *Collaborator*, and *Manager*. Close reference to the CanMEDS roles competency lists assisted in the development of specific case scenarios and items for the checklists to ensure that core competencies were addressed and assessed. The checklists developed were functional and reusable, but the items required more consistency across stations, as they were designed to meet the specific context for each scenario. Further development of the checklists is a lengthy process and requires considerable collaboration and inputs from clinical experts. Although these initial findings demonstrate the potential of using the CITE testing format for the assessment of intrinsic competencies, more generic types of checklists may also improve the correlations found on similar CanMEDS roles between stations.

The within-station reliability for the checklists used was satisfactory given the reliability coefficients above *α*=0.70, indicating high internal consistency within stations (26). There were, however, no clear patterns in terms of correlations between stations and across CanMEDS roles. Furthermore, general communication scores were found not to correlate well between stations. Similarities in the themes between the cases could account for between-station correlation across CanMEDS roles or stations. The *Health Advocate* and *Communicator* scores were highly correlated showing that these competencies maybe be strongly related domains in medicine. For example, in order to be a competent *Health Advocate* for patients, one must also be a similarly competent *Communicator* which makes intuitive sense. Insignificant correlations between other CanMEDS roles and stations could be evidence that the other CanMEDS roles are more distinct from each other as demonstrated by the results found with the case scenarios from this study.

Similar to the results of other studies that used OSCEs in pediatric and orthopedic residents described earlier (13, 15), our study only showed a difference with respect to *Professional* competence performance on the OSCE between senior and junior residents. It is also worth noting that internal medicine residents had lower scores on the *Professional* CanMEDS role compared with non-internal medicine residents. Furthermore, the significant results seen across cases 1, 3, and 5 may be useful in differentiating between residents who belong to other programs that are not based on internal medicine such as family medicine, general surgery, and so on.

The *Communicator* role and its communication-related competencies have been more widely tested using a variety of assessment methods, including the OSCE. Previous studies show the need for larger studies and more scrutiny in SP and rater training before the assessment can be considered reliable (10, 11, 27). Given our results showed a large correlation between general communication and the primary and secondary CanMEDS roles within each station, further work should look at correlation across stations.

In terms of our validity assessment, *content evidence* was achieved by seeking expert review and utilizing an assessment framework such as the CanMEDS roles. Our study fell short in terms of gathering *response process evidence* in that although examiners were met with before the exam to go over their respective cases, we have no evidence of how well the raters’ actions related to the intended measurement construct. Although the OSCE was videorecorded for the CITE, we are yet to determine the inter-rater reliability of the checklists as we only had one examiner per station, and we would require several examiners to watch the videorecorded cases to determine the inter-rater reliability. Regarding *internal structure evidence*, we have shown satisfactory evaluation of how well assessment items aligned with the overall construct by showing high reliability within stations.

We did not have any strong evidence from an item analysis as there were no correlations between the same CanMEDS role from one station to another or even correlations between general communication scores across stations. A factor analysis could not be conducted with the current data, given the small sample size and the large number of checklist items.

In terms of validity evidence pertaining to *relations with other variables*, our results showed possible differences between internal medicine residents and non-internal medicine residents for some of the CanMEDS roles and stations. Unlike Dwyer and colleagues (11), we did not explore the relationship of the CITE scores with that of other measurements such as ITERS or other forms of assessment.

Lastly, because the CITE was used to assess residents as part of a new formative assessment approach, we are also lacking *consequences evidence* as our results have had no known impact that may have led to decisions or actions that influenced outcomes. Although the residents were all provided with immediate verbal feedback after the OSCE and a performance report several weeks after the OSCE, we do not know whether their results influenced their learning or training in any way.

Our study had several limitations: 1) we do not have any evidence of inter-rater reliability; 2) we lack strong evidence of relationships with other variables such as PGY level or other assessment tools; and 3) we do not know of any evidence of any impact that the CITE may have had on the residents’ learning, performance, or practice. Furthermore, given that this was a pilot study, we did not have the expected minimum levels of performance for junior and senior residents for the CITE. Strengths of our study included 1) having a larger sample size compared with previous studies; 2) using a thorough and detailed process for developing cases and checklists; and 3) videorecording the stations for future research pertaining to quality assurance and inter-rater reliability.

We have shown that an OSCE measuring intrinsic CanMEDS roles is feasible; however, further development of our cases and checklists will be required to ensure that we can establish evidence for construct validity. We provide a model of how to develop an OSCE to measure intrinsic CanMEDS roles that educators may adopt as residency programs move into the CBME format.
